# Interpreting Evolving Evidence in Alzheimer’s Disease: Implications for Dementia Clinicians

**DOI:** 10.1192/bjp.2026.10750

**Published:** 2026-08-05

**Authors:** Ivan Koychev, James Rowe, Jay Amin, Dag Aarsland, Hilary Archer, Robert Barber, Alistair Burns, Elizabeth Coulthard, Charlotte Deasy, Ross Dunne, Nick Fox, Sara Humphrey, Josie Jenkinson, Danielle Jones, Sean Kennelly, Farooq Khan, M Santhana Krishnan, Paresh Malhotra, Brady McFarlane, Bernadette McGuinness, Catherine Mummery, Catherine Pennington, John Richard Perry, Gosia Raczek, Jill Rasmussen, Craig Ritchie, John Paul Taylor, Benjamin R Underwood, Ash Venkataraman, Vanessa Raymont

**Affiliations:** 1 Imperial College London; 2 University of Cambridge; 3 University of Southampton; 4 Hampshire and Isle of Wight Healthcare NHS Foundation Trust; 5 King’s College London; 6 North Bristol NHS Trust; 7 Newcastle University; 8 University of Manchester; 9 Oxford Health NHS Foundation Trust; 10 University of Bristol; 11 Hillingdon Hospitals NHS Foundation Trust; 12 UK Brain Health Coalition; 13 Greater Manchester Mental Health NHS Foundation Trust; 14 UCL Institute of Neurology, Dementia Research Centre; 15 Department of Applied Dementia Studies, University of Bradford; 16 Cleveland Clinic London; 17 Surrey and Borders Partnership NHS Foundation Trust; 18 Tallaght University Hospital; 19 University of Chester; 20 Birmingham and Solihull Mental Health NHS Foundation Trust; 21 Tees Esk and Wear Valleys NHS Foundation Trust; 22 Southern Health NHS Foundation Trust; 23 Queen’s University Belfast; 24 NIHR UK Dementia Trials Network; 25 University of Edinburgh; 26 Imperial College Healthcare NHS Trust; 27 West London NHS Trust; 28 psi-napse; 29 Scottish Brain Sciences; 30 University of St Andrews; 31 King’s College London, IoPPN; 32 South London and Maudsley NHS Foundation Trust; 33 University of Oxford

The recent Cochrane systematic review of amyloid-targeting therapies for early-stage Alzheimer’s disease (1) has understandably received a great deal of public interest. Unfortunately, its interpretation risks misrepresenting the current state of therapeutic development in this field.

The central concern lies in the aggregation of data from very different agents. The meta-analysis pools early, largely negative trials with later agents with different targets. The later agents have demonstrated efficacy in larger phase 3 trials, and received regulatory approval from bodies including the MHRA, FDA and EMA. While systematic reviews are, by definition, inclusive, the absence of meaningful stratification by developmental phase, mechanism, or trial design introduces a substantial interpretive limitation. In addition, the conclusion of the review that “amyloid removal” doesn’t produce clinical benefits does not fit with the stated aim of the review which was to test whether the monoclonals produced clinical benefit, when some of the monoclonals assessed had absolutely no effect on amyloid.

Alzheimer’s disease trials have also evolved in design. Early trials were often conducted without biomarker confirmation, with suboptimal dosing, or with incomplete understanding of target engagement. Later-phase trials of currently approved therapies differed in patient selection, methodological rigour, and biological precision. Treating these distinct trials as directly comparable conflates early exploratory failure with later positive phase 3 outcomes. Although the review also examined the subgroup of agents that have subsequently received regulatory approval, pooled estimates alone may not fully capture differences in biological target engagement, trial methodology, and duration of follow-up that are relevant to interpretation of treatment effects.

For clinicians working with people with or at risk for dementia, this distinction is critical. Decision-making increasingly depends on nuanced interpretation of evolving evidence, particularly where disease-modifying therapies are concerned. Analyses that do not adequately distinguish between different generations of trials and therapeutic approaches risk obscuring clinically relevant effects that have met contemporary regulatory standards.

The Cochrane review also discusses the meaningfulness of the drugs’ benefits. The assessment of meaningfulness should incorporate the perspectives of people living with Alzheimer’s disease and their families, while remaining grounded in outcomes directly supported by randomised trial evidence.

The broader implications are also significant. Cochrane reviews carry considerable weight in shaping public discourse and policy. It is therefore unsurprising that patient organisations and charities have responded with caution. There is a risk that such analyses may be used by payers or decision-makers as justification to restrict access to approved treatments, particularly if the conclusions do not sufficiently distinguish between exploratory and confirmatory phases of drug development.

The review provides a valuable synthesis of available evidence, but its interpretation may understate important differences between therapeutic generations and patterns of target engagement. Critical evaluation of amyloid-targeting therapies is essential and entirely appropriate. However, analyses should distinguish between agents that failed to engage their biological target and those that demonstrated substantial amyloid removal and subsequently received regulatory approval. Greater attention to biological target engagement, trial design, and therapeutic generation would provide a more clinically informative synthesis of the available evidence.

The field would also benefit from full publication of long-term extension analyses, including comparisons between early- and delayed-treatment groups, to clarify the durability and cumulative clinical impact of treatment effects.
